# The neurobiology of treatment-resistant schizophrenia: paths to antipsychotic resistance and a roadmap for future research

**DOI:** 10.1038/s41537-019-0090-z

**Published:** 2020-01-07

**Authors:** Steven G. Potkin, John M. Kane, Christoph U. Correll, Jean-Pierre Lindenmayer, Ofer Agid, Stephen R. Marder, Mark Olfson, Oliver D. Howes

**Affiliations:** 1 University of California at Irvine, Irvine, CA USA; 2 Donald and Barbara Zucker School of Medicine at Hofstra/Northwell, Department of Psychiatry and Molecular Medicine, Hempstead, NY USA; 3 The Zucker Hillside Hospital, Glen Oaks, NY USA; 4 The Feinstein Institute for Medical Research, Psychiatric Neuroscience Center of Excellence, Manhasset, NY USA; 5 Charité Universitätsmedizin, Department of Child and Adolescent Psychiatry, Berlin, Germany; 6 Department of Psychiatry, New York University School of Medicine, New York, NY USA; 7 Centre for Addiction and Mental Health, Department of Psychiatry, University of Toronto, Toronto, ON Canada; 8 The Semel Institute for Neuroscience at UCLA, Los Angeles, CA USA; 9 The VA Desert Pacific Mental Illness Research, Education, and Clinical Center, Los Angeles, CA USA; 10 Department of Psychiatry, Columbia University Irving Medical Center, New York, NY USA; 11 King’s College, London, UK; 12 MRC London Institute of Medical Sciences, Imperial College, London, UK

**Keywords:** Schizophrenia, Schizophrenia

## Abstract

Treatment-resistant schizophrenia (TRS), the persistence of positive symptoms despite ≥2 trials of adequate dose and duration of antipsychotic medication with documented adherence, is a serious clinical problem with heterogeneous presentations. TRS can vary in its onset (at the first episode of psychosis or upon relapse), in its severity, and in the response to subsequent therapeutic interventions (i.e., clozapine, electroconvulsive therapy). The heterogeneity of TRS indicates that the underlying neurobiology of TRS may differ not only from treatment-responsive schizophrenia but also among patients with TRS. Several hypotheses have been proposed for the neurobiological mechanisms underlying TRS, including dopamine supersensitivity, hyperdopaminergic and normodopaminergic subtypes, glutamate dysregulation, inflammation and oxidative stress, and serotonin dysregulation. Research supporting these hypotheses is limited in part by variations in the criteria used to define TRS, as well as by the biological and clinical heterogeneity of TRS. Clinical trial designs for new treatments should be informed by this heterogeneity, and further clinical research is needed to more clearly understand the underlying neurobiology of TRS and to optimize treatment for patients with TRS.

## Introduction

**Fig. 1:**
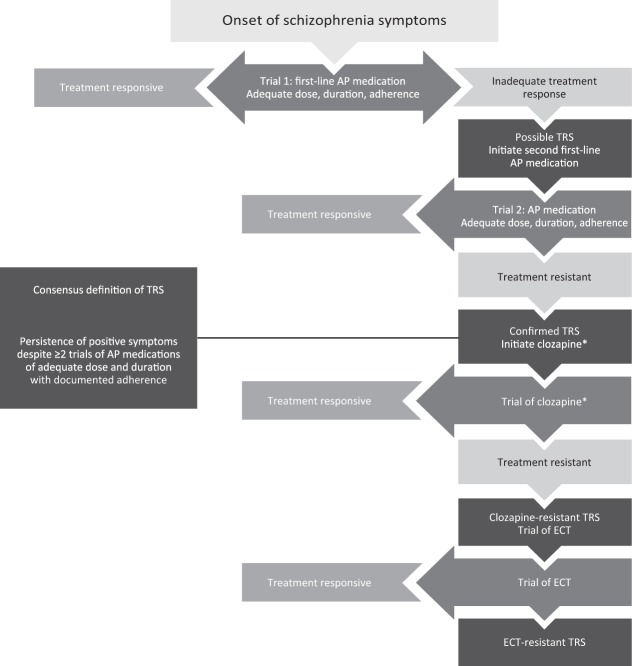
Clinical path to confirmation of TRS. AP antipsychotic, ECT electroconvulsive therapy, TRS treatment-resistant schizophrenia. ^*^If a trial of clozapine was not previously completed.

Treatment-resistant schizophrenia (TRS) has been defined as the persistence of symptoms despite ≥2 trials of antipsychotic medications of adequate dose and duration with documented adherence.^1,2^ TRS occurs in up to 34% of patients with schizophrenia.^3–5^ Although persistent symptoms may be negative or cognitive,^1^ persistence of positive symptoms is generally one of the defining features of TRS.^6^ However, the failure of serial antipsychotic medication trials is not sufficient to define TRS, as other potential causes of persistent symptoms need to be excluded as well (Table 1). A typical clinical pathway to identifying patients with TRS is shown in Fig. 1.

**Table 1:** Comparison of treatment-resistant schizophrenia vs pseudo-resistance.^8,11^

TRS	Pseudo-resistance
Cause: pharmacodynamic factors	Cause: clinical or pharmacokinetic factors
● Adequate dose and duration of antipsychotic treatment	● Incorrect diagnosis
● Associated with early age of onset and long duration of illness	● Inadequate dose or duration of antipsychotic treatment
● Biological differences from treatment-responsive schizophrenia	● Insufficient plasma levels of antipsychotic medication
	● Poor treatment compliance or adherence
	● Adverse events or comorbid medical conditions masking response
	● Substance use triggering psychosis

*TRS* treatment-resistant schizophrenia

TRS may present from the first episode of psychosis^3,4,7^ or may develop later in the disease progression. Later onset of treatment resistance may be preceded by relapses,^8–10^ which in part may result from medication nonadherence or discontinuation.^2^ However, it is critical to differentiate true TRS from pseudo-resistance (i.e., when a patient appears resistant, but treatment is inadequate rather than ineffective).^8,11^ TRS results from lack of response to adequate exposure to medication with no confounding factors (Table 1), whereas pseudo-resistance may occur as a result of medication nonadherence, insufficient plasma levels of a medication, inadequate dosage or duration of treatment, misdiagnosis, adverse events of a treatment masking a response, or the presence of confounding psychiatric or medical comorbidities.^8,11,12^

Outcomes for patients with treatment resistance may be improved if identification of TRS occurs earlier in the course of disease rather than after a long duration of untreated psychosis.^13,14^ Early identification of TRS may allow for early introduction of clozapine, the only approved antipsychotic for TRS.^12,15^ There is some evidence suggesting that a trial of clozapine may be warranted after even one failure of a non-clozapine antipsychotic course of treatment,^16–18^ although this requires further research. Up to 60% of patients with treatment resistance will not respond even to clozapine.^6,19^ Although a wide variety of medication augmentation strategies have been tried, there is a lack of strong evidence regarding the efficacy of such strategies; thus, patients with TRS may have limited treatment options.^20,21^ Electroconvulsive therapy (ECT) was found to be helpful for some patients with clozapine-resistant schizophrenia.^22,23^

There may be underlying biological differences between patients with TRS and patients with treatment-responsive schizophrenia.^24^ Current hypotheses for the biological basis of TRS focus on differences in the functioning of dopaminergic pathways (i.e., supersensitivity or hyper-, normo-, or hypodopaminergic schizophrenia) or changes in glutamate or other neurotransmitter pathways. These theories are not mutually exclusive, with several pathways converging and possibly contributing to the neurobiology of TRS. Elucidating the underlying pathophysiology of TRS may aid in better treatment selection and inform development of future treatments.^8,25^ Furthermore, additional research identifying biomarkers of clozapine resistance or response is needed, as well as new treatments for clozapine-resistant schizophrenia.

The goal of this review is to examine advances in our understanding of the underlying neurobiology of TRS as it relates to positive symptoms, that is, symptoms that were not responsive to antipsychotic treatment from illness onset or that were previously, but are no longer, responsive to antipsychotic treatment. Treatment-resistant negative and cognitive symptoms are of great clinical relevance, but the underlying pathophysiology mechanisms are not well understood and more research is needed to identify appropriate treatments for these domains; negative and cognitive symptoms, therefore, are not discussed further in this review. This review examines potential neurophysiological and molecular mechanisms of TRS related to positive symptom treatment targets. Connectivity and volumetric data may provide further insight into the neurobiological mechanisms of TRS but are not considered here because they are less likely to be relevant to the development of new pharmacologic treatments.

## Neurobiology of TRS

TRS may develop via one of several neurobiological pathways. Several lines of evidence point to dopamine and glutamate dysfunction in the development of TRS, although there is some evidence that serotonin pathway dysfunction may also play a role.^8,26^ One theory of TRS has been the dopamine supersensitivity psychosis (DSP) hypothesis. This hypothesis, explaining TRS in patients who were responsive to antipsychotic treatment at illness onset, posits that continuous blockade of dopamine receptors by antipsychotic medications leads to dopamine supersensitivity, causing TRS.^27,28^ However, the DSP hypothesis is not universally accepted, and clinical data do not consistently support it. This review considers the evidence for and against the DSP hypothesis and presents several other potential pathways and neurobiological mechanisms that may lead to TRS.

## Dopamine supersensitivity hypothesis

The DSP hypothesis was first proposed by Chouinard et al. in 1978,^27^ and subsequent work has acknowledged this hypothesis as a potential etiology for some cases of TRS. The main known mechanism of action for antipsychotic medication is dopamine D2 receptor (DRD2) blockade.^29–31^ The dopamine supersensitivity theory proposes that continuous blockade of DRD2 results in dopaminergic changes that lead to breakthrough symptoms that are no longer effectively treated by the initial dose of antipsychotic medication.^27,28,32–35^ This chain of events is proposed to lead to the need for increasing doses of antipsychotic drugs to control symptoms, and rapid relapse during withdrawal or dose reduction of an antipsychotic medication, or as tolerance develops to previously therapeutic doses of antipsychotic medication with continuous treatment.^36^

**Fig. 2:**
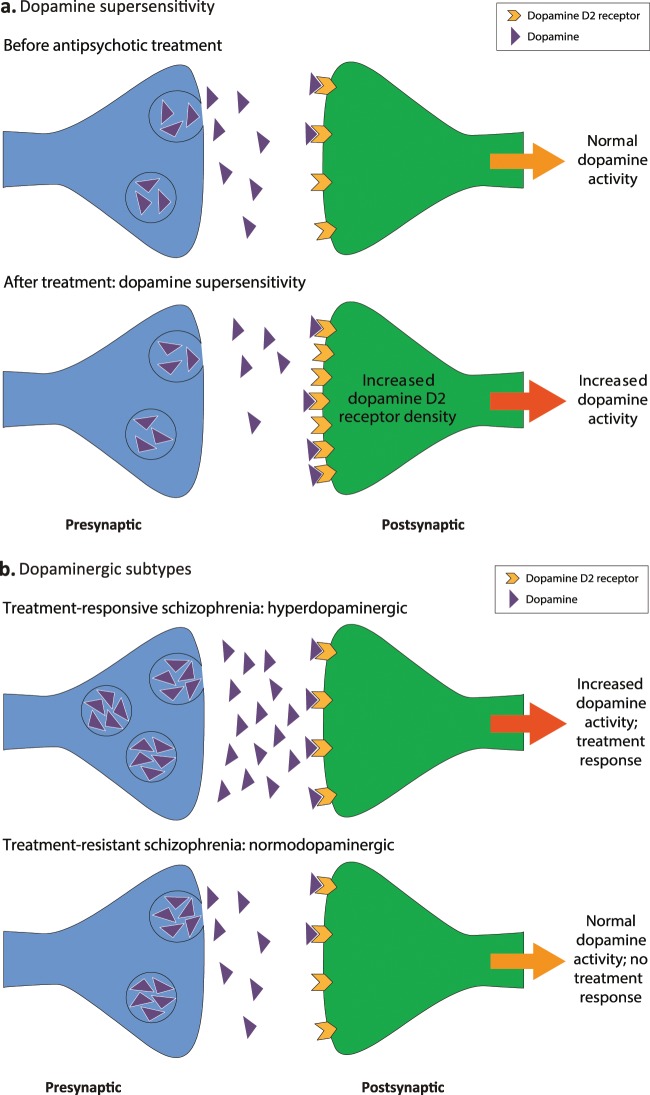
Dopaminergic pathways to treatment resistance. (**a**) Dopamine supersensitivity and (**b**) dopaminergic subtypes.

The dopaminergic changes following continuous receptor blockade with an antipsychotic medication are proposed to involve increases in DRD2 receptor density (Fig. 2a).^33,37^ In turn, increases in antipsychotic medication doses to control breakthrough symptoms are thought to lead to further increases in DRD2 density, resulting in increased dopamine supersensitivity, and consequently, the reemergence of symptoms.^33,37^ This implies that TRS is related to duration of antipsychotic treatment; however, positive symptoms of schizophrenia decrease with age and do not increase as the DSP hypothesis implies.^38^ The emergence of tardive dyskinesia is also proposed as a clinical characteristic reflecting dopamine supersensitivity,^33^ although tardive dyskinesia is not typically considered a feature of treatment resistance.^1^ The dopamine supersensitivity hypothesis has generally focused on DRD2 receptor changes but could also involve presynaptic changes,^39^ albeit these do not seem to be marked with second-generation antipsychotics.^40^

A survey from Suzuki et al. found that 70% of patients with TRS showed one or more clinical characteristic consistent with the DSP hypothesis. However, the criteria for DSP used in this study included presence of either tardive dyskinesia or rebound psychosis,^32^ and reliance on medical records rather than systematic research assessments introduces uncertainty into the classification of antipsychotic treatment and clinical status results. Similarly, an observational study testing the effectiveness of risperidone long-acting injection in patients with TRS found that 61 of 94 patients (65%) also had clinical characteristics proposed to be due to DSP; although again, the presence of tardive dyskinesia was sufficient for a designation of DSP.^41^ In patients with TRS and proposed DSP, risperidone long-acting injection improved scores on the Brief Psychiatric Rating Scale by ≥20%, a significantly greater improvement than seen in patients with TRS but without proposed DSP.^41^ This finding suggests that patients meeting criteria for both DSP and TRS may be better treatment responders than patients meeting criteria for TRS but not DSP when the delivery method of antipsychotic medication promotes stable blood levels within an optimal therapeutic window.^41^ However, it is important to note that identification of DSP is only possible if a patient previously responded to antipsychotic treatment, indicating that only patients who developed TRS after initial response to antipsychotics fall into this category.^33^ A further caveat to the survey and the long-acting injectable study^41^ is that, in both studies, patients could be classified as having DSP if they displayed tardive dyskinesia without showing breakthrough positive symptoms. Although tardive dyskinesia is related to dose and duration of antipsychotic treatment, it is not equivalent to treatment resistance and is generally not considered a required diagnostic feature of TRS.^32,41,42^

One prediction of the dopamine supersensitivity hypothesis is that patients who have received prolonged antipsychotic treatment (i.e., who would be anticipated to have developed supersensitivity) will relapse rapidly on antipsychotic withdrawal. However, relapse profiles, as well as clinical characteristics, are generally similar in patients with longer and shorter treatment durations.^43^ From a clinical perspective, relapse despite full adherence after achieving remission does occur, as can best be seen in relapses on long-acting injectable antipsychotic medications used after oral antipsychotic stabilization.^44^ Approximately 18% of patients receiving long-acting injectable antipsychotic medications (for which adherence is ensured) relapse within 1 year,^45,46^ although it is difficult to determine whether these patients were truly in remission at baseline. The results of these studies examining relapse of patients on long-acting injectable antipsychotic medications are not supportive of the dopamine supersensitivity hypothesis.

If DRD2 receptors are upregulated or otherwise supersensitive because of long-term antipsychotic treatment, in line with the DSP hypothesis, and if medication is abruptly withdrawn, high levels of unblocked receptors may interact with endogenous dopamine to rapidly increase psychosis. In contrast, if medication is gradually withdrawn, DRD2 receptors could downregulate in advance of complete removal of blockade, allowing the supersensitivity to resolve. Hence, a prediction of the DSP hypothesis is that relapse rates should be higher in patients who abruptly, rather than gradually, withdraw from antipsychotic treatment. However, a meta-analysis failed to find differences in relapse rates between patients gradually or abruptly discontinuing antipsychotic medications.^47^ A recent study found that relapse rates were higher in patients withdrawn from long-acting injectable paliperidone palmitate (i.e., gradual withdrawal of antipsychotic medication) and switched to placebo treatment compared with those receiving maintenance treatment (hazard ratio, 3.60; 95% confidence interval, 2.45–5.48).^45^ However, there was no significant difference in relapse symptom severity between patients who experienced a relapse while continuing treatment and those who relapsed upon medication withdrawal, indicating that relapse upon withdrawal is not clinically different from illness recurrence; abrupt return of psychotic symptoms was also common in both groups.^45^

If upregulation of DRD2 receptors occurs with prolonged use of antipsychotic medication, then patients would be expected to demonstrate tolerance, necessitating higher doses over time. A recent study found that the dosage of antipsychotic medication necessary to treat a relapse of psychosis was significantly higher than the dosage that had been necessary to treat first-episode psychosis, lending support to the tolerance aspect of the DSP hypothesis.^48^ However, there are other possible explanations for the need for an increased dose: each episode of psychosis may be evidence of disease progression, necessitating higher doses of medication to achieve symptom relief.^48^ A recent study found that patients took longer to respond to treatment after a relapse of psychosis than for a first episode, even after adjustment for antipsychotic medication dose.^10^ Duration of illness (>10 years vs >5 years) was a significant predictor of relapse, even with the ensured adherence of treatment with a long-acting injectable antipsychotic medication, suggesting that disease progression of schizophrenia affects response to treatment.^46^ Finally, DRD2 receptor upregulation occurs on a much shorter timescale, on the order of weeks rather than years,^27^ making DSP an unlikely candidate for the mechanism of treatment resistance in the study by Alphs and colleagues.^46^

Studies in a rat model of schizophrenia have investigated the effects of antipsychotic treatment on the dopamine system. Loss of gamma-aminobutyric acid (GABA) interneurons during adolescence in the methylazoxymethanol acetate (MAM) model led to hyperactivity of midbrain dopaminergic neurons, consistent with findings in patients with schizophrenia.^49^ Treatment with a positive allosteric modulator of the α5 subunit of GABA-A receptor, which restores some of the inhibitory capabilities to brain regions that regulate dopaminergic neurons, ameliorated schizophrenia-like symptoms in MAM-treated rats.^50^ However, pretreating MAM-treated rats with haloperidol, an antipsychotic DRD2 antagonist, for 3 weeks and then discontinuing it before initiation of the GABA modulator blocked these therapeutic effects of the GABA modulator.^35^ This finding suggests that even temporary dopamine receptor blockade can alter the effects of future treatments on the dopamine system, even when they do not directly act on dopamine receptors.

As previously mentioned, an important caveat to the DSP hypothesis is that it accounts only for cases of TRS that emerge after an initially successful treatment with an antipsychotic medication.^32,36^ Thus, it is likely that TRS apparent at the first episode of schizophrenia is neurobiologically distinct from TRS that develops over time.^10^

## Hyperdopaminergic and normodopaminergic subtypes hypothesis

Increased striatal dopamine synthesis and release capacity in vivo are linked to psychotic relapse and the development of the first psychotic episode,^51–53^ with large effect size elevations on meta-analysis.^54^ However, it has been proposed that not all patients with schizophrenia display striatal hyperdopaminergic activity and that some patients with TRS demonstrate normal dopamine regulation or even hypodopaminergic activity (Fig. 2b).^25^ Evidence for this hypothesis comes from positron emission tomography studies showing that dopamine synthesis capacity in the striatum is significantly higher in vivo in patients with treatment-responsive schizophrenia relative to patients with TRS, who, in turn, have a dopamine synthesis capacity similar to healthy controls.^24,53,55^

Dopamine synthesis capacity in the striatum has been shown to be lower in patients with TRS who are clozapine-responsive compared with healthy controls or patients without TRS.^56^ An important issue is whether these differences between treatment-responsive and treatment-resistant patients are present from illness onset or not. A recent study in first-episode patients found evidence that this was the case (i.e., differences present from illness onset), with elevated striatal dopamine synthesis capacity in first-episode patients who went on to respond to antipsychotic treatment, but unaltered dopamine synthesis capacity in first-episode patients who did not respond to antipsychotic treatment.^53^ This finding suggests that there may be dopaminergic subtypes of schizophrenia of treatment resistance from illness onset. Notwithstanding these lines of in vivo evidence,^53,56^ one important issue is whether dopamine differences between TRS and responsive patients are categorical, as proposed by the subtype hypothesis, or whether they are a matter of degree, as suggested by correlations between dopamine synthesis capacity and symptom change with antipsychotic treatment.^53^ Further cross-sectional and longitudinal studies are needed to address this issue (see research roadmap below).

## Genetic variants that may contribute to schizophrenia or TRS

Possible associations between several dopamine-related polymorphisms and TRS have been investigated. A combination of dopamine transporter (DAT-40 base pair variable number of tandem repeat [DAT-VNTR]) and serotonin transporter (SERT-VNTR intron 2 [SERT-in2]) polymorphisms has been associated with TRS.^57^ A study of two single-nucleotide polymorphisms (SNPs) in the dopamine-degrading enzyme catechol-*O*-methyltransferase (COMT) demonstrated that a higher-activity haplotype was protective against TRS in women but not men.^58^ Genetic polymorphisms in DRD2 may also affect a patient’s risk of developing DSP TRS or the likelihood that a patient will respond to non-clozapine antipsychotic medications.^36,59^ However, another study showed no association between TRS and SNPs in DRD1, DRD2, DRD3, or COMT.^60^ A study of copy number variations (CNVs) in a Japanese population observed that patients with clinically significant de novo CNVs were more likely to have TRS, perhaps indicating genetic instability.^61^ A genome-wide association screen in patients with schizophrenia found an association between variation in genes related to targets of some antipsychotic medications and TRS, as defined by a lack of response to standard treatments.^62^ The gene sets and individual genes most associated with antipsychotic treatment targets included N-methyl-D-aspartate (NDMA)-relevant genes and GRIN2a, in particular.^62^ Singleton deleterious variants found in genes targeted by antipsychotic medications were enriched in individuals with TRS, as defined by the presence of a clozapine prescription.^62^

Notably, these genes were not all related to dopaminergic receptors, indicating that development of TRS may occur through multiple pathways.^62^ A study comparing gene expression among patients with TRS, patients with treatment-responsive schizophrenia, and healthy controls found no difference between the groups with schizophrenia in the expression of 13 candidate genes, including COMT.^63^ Although there are inconsistencies and variability among the results of some of these highlighted studies, several candidates for the biological mechanisms or subtypes of TRS require further research. Additional studies are needed to further characterize these candidate biological mechanisms and to identify new treatment targets.

## Glutamate hypothesis

Although dopamine dysregulation clearly contributes to the symptoms of schizophrenia in many patients, the failure of antipsychotic dopamine blockade to control symptoms in some patients implies that other neurotransmitters likely play a role in the etiology of TRS, as suggested by the subtype hypothesis.^64^ The most prominent hypothesis for the involvement of other neurotransmitter systems in the neurobiology of schizophrenia is the glutamate hypothesis, which suggests that dopamine hyperactivity is downstream of GABA and glutamate dysregulation.^26^ According to the glutamate hypothesis, NMDA receptor dysfunction on GABA interneurons results in disinhibition of cortical or hippocampal glutamate neurons projecting to the basal ganglia. This hyperactivation of glutamate neurons, in turn, stimulates activity of dopaminergic projections from the midbrain to the striatum, resulting in the positive symptoms of schizophrenia.^64–66^ This theory is supported by observations that patients with schizophrenia show an exacerbation of positive symptoms after administration of phencyclidine (PCP), an NMDA receptor antagonist, and that PCP mimics the positive effects of schizophrenia in healthy volunteers.^67,68^

There is evidence that abnormalities in glutamate regulation may specifically play a role in TRS.^24,25,69^ Neuroimaging studies have measured brain glutamate levels in patients with schizophrenia in comparison with healthy controls.^24,70^ Glutamate levels in the anterior cingulate cortex were higher in patients with TRS compared with healthy controls or patients with schizophrenia who were treatment responsive.^24,70^

Another neuroimaging study measured levels of glutamate as well as glutamate + glutamine (Glx) in several brain regions in patients with TRS treated with clozapine in comparison with patients who were first-line antipsychotic treatment responders, patients with TRS unresponsive to clozapine, and healthy controls.^69^ Glx is the precursor for glutamate.^69^ Glx but not glutamate levels were higher in the putamen of patients with clozapine-responsive TRS than in first-line responders or patients with clozapine-nonresponsive TRS.^69^ This difference between groups could potentially be related to differences in the activity of the enzyme that converts Glx to glutamate or to a problem with Glx reuptake, resulting in excess Glx.^69^ Although the precise mechanisms of action of clozapine are unknown, this work may support evidence for a contribution of a glutamate-related mechanism of action.^69^

Indirect evidence for glutamate’s role in clozapine-responsive TRS is supported by studies examining the effect of clozapine on the glutamatergic system.^71^ Clozapine has high affinity for DRD4, and in a rat model, blockade of DRD4 upregulated α-amino-3-hydroxy-5-methyl-4-isoxazoleproprionic acid (AMPA) receptors, improving glutamatergic transmission.^72,73^ Clozapine also increases release of L-glutamate and D-serine in rat frontal cortex, which can result in upregulation of NMDA receptors.^71^ If NMDA receptors are hypofunctional in TRS, it is possible that clozapine could partially compensate for this dysfunction.^73^ In a study examining D- and L-serine levels in patients with TRS before and after clozapine treatment, patients with TRS had significantly lower D-serine levels before clozapine treatment than did healthy controls, a difference that disappeared after clozapine treatment.^74^ Similarly, the D-/L-serine ratio was significantly lower in patients with TRS before clozapine treatment but not significantly different from healthy controls after clozapine treatment, providing further support to the hypothesis that clozapine’s mechanism of action may be related to regulation of a dysfunctional glutamatergic pathway.^74^ Glycine opens NMDA channels and thus could counteract a hypoglutamatergic state; however, in a double-blind study of patients with TRS taking 30 g of glycine adjunctive to optimal doses of clozapine, patients taking only clozapine demonstrated significant improvement in positive symptoms.^75^ The multicenter CONSIST study also found no beneficial effects of high-dose glycine or glutamatergic agents on negative or cognitive schizophrenia symptoms.^76^ Additionally, data from phase 3 clinical trials of adjunctive bitopertin, a glycine reuptake inhibitor, showed no statistical difference from placebo in positive or negative symptoms.^77,78^ Nevertheless, these studies were not specifically in TRS, highlighting that it would be useful to test this mechanism in this patient population. Other drug targets and mechanisms in the glutamate pathway should also be explored.

In patients with TRS not responding to clozapine, concomitant use of glutamate release inhibitors such as lamotrigine^73^ or topiramate^79^ may increase treatment response; however, the quality of studies has been mixed, and the efficacy of various pharmacological augmentation strategies in patients with clozapine-resistant schizophrenia remains unclear.^21^ Further high-quality randomized clinical trials are needed to establish the effectiveness of these glutamatergic augmentation strategies and to identify patient subgroups that have a higher likelihood of responding to specific pharmacological combination or augmentation strategies.^21^

## Inflammation and oxidative stress

Neuroinflammation early in life followed by chronic overactivation has been hypothesized to contribute to the etiology of schizophrenia; it is possible that high levels of inflammation play a role in treatment resistance as well.^80,81^ Specific immune-inflammatory cytokine profiles have been associated with both treatment-responsive schizophrenia and TRS^82^; levels of these biomarkers can be present from illness onset and thus do not represent a downstream effect of psychosis.^81^

Oxidative stress, as measured by lipid peroxidation, was elevated in patients with TRS compared with patients with treatment-responsive schizophrenia and healthy controls.^83^ It is possible that oxidative stress–related neuronal damage also contributes to TRS. Individuals with gene deletions in the antioxidant glutathione S-transferase family may have an increased risk of TRS compared with healthy controls.^84^ However, this study was limited by the lack of a treatment-responsive schizophrenia control group,^84^ and more research is needed to determine the role of oxidative stress in TRS.

## Serotonin

Serotonin plays a complex role in modification of dopamine neurotransmission and is thought to contribute to the neurobiology of schizophrenia.^85,86^ There is evidence of lower levels of serotonin 2A receptors (and higher levels of serotonin 1A receptors) postmortem with moderate to large effect sizes in schizophrenia in general, although imaging findings are inconsistent and treatment resistance has received little attention.^87^ Notwithstanding this, polymorphisms in serotonin receptors have been associated with response to clozapine.^88^ Modulators of serotonin have shown some efficacy in improving negative symptoms as augmentation therapy in combination with antipsychotics medications,^89^ but higher-quality studies are needed to confirm these findings.^21^

## Electroconvulsive therapy

Concomitant use of clozapine and ECT may also be valuable as an augmentation strategy in patients who do not respond to clozapine alone, raising additional questions about the underlying mechanisms of treatment resistance.^22,23^ A prospective study found that half of patients with TRS not responding to clozapine treatment alone demonstrated ≥40% improvement on the Brief Psychiatric Rating Scale psychotic symptom subscale when concomitantly treated with ECT.^23^ A meta-analysis examining the efficacy of ECT augmentation confirmed that it is both effective and safe in patients with clozapine-resistant TRS.^22^ The efficacy of this strategy may provide insight into the mechanisms of TRS and requires further study.

## Limitations of evidence for the possible mechanisms of TRS

The evidence considered in our review is limited by several factors, including variation in the criteria used to define TRS, small sample sizes of many studies, and the fact that few studies differentiate between initial and later onset TRS.^1^ Consequently, there is not a universally accepted or definitive mechanism for TRS. There is also heterogeneity among patients with schizophrenia, including those with TRS, in their clinical characteristics, response to treatment, and underlying neurobiology.^90^ It is likely that there are several distinct subgroups and neurobiological profiles of patients with TRS; further work is needed to differentiate these subgroups, define their characteristics and possible biomarkers, and study their individual response patterns to treatment. Among patients with TRS, there are also important clinical differences, most strikingly between patients who respond to clozapine and those who do not. A key future direction is to develop biomarkers to predict TRS early in the course of illness to identify the neurobiological mechanisms underlying TRS and guide treatment selection. Candidate serological biomarkers for TRS associated with schizophrenia treatment response could include decreased brain-derived neurotrophic factor (BDNF) and levels of soluble interleukin (IL)–2 receptor (sIL-2R), IL-6, and IL-1 receptor antagonist (IL-1RA).^81,91,92^ Other candidate biomarkers could include normal striatal dopamine synthesis despite a schizophrenia diagnosis and resting state striatal connectivity.^24,93,94^ Biomarkers that predict treatment resistance or clozapine response will likely be difficult to implement if they are very expensive or difficult to obtain (e.g., lumbar puncture, neuroimaging), but these factors should be weighed against the high cost of TRS.^95^ It is likely that a combination of biomarkers and patient clinical information will improve prediction of TRS and medication response.^95,96^

## Research roadmap

**Fig. 3:**
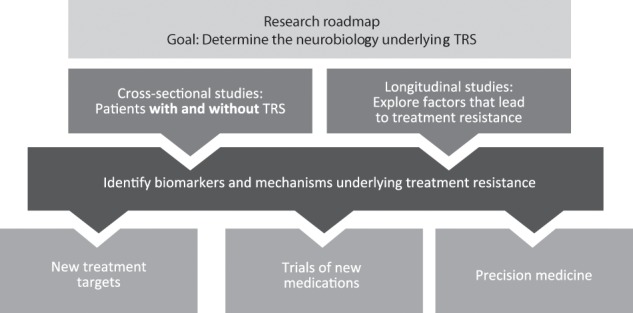
Clinical research priorities for TRS. TRS treatment-resistant schizophrenia.

The neurobiological diversity of schizophrenia as exemplified by TRS suggests that clinical trial strategies need to be informed by both the biology and diverse clinical presentation of the individual patient.^25^ The cross-sectional diversity of the neurobiology of TRS, coupled with the clinical evidence for TRS being present from illness onset in some patients and developing over time in others, indicates that there need to be biological studies of phase-specific underlying mechanisms of TRS. Such studies include examining TRS in first-episode psychosis, TRS in established schizophrenia and TRS emerging in older age patients, and longitudinal studies that investigate biological changes in the dopamine and other systems during the course of illness. Another important consideration is whether the biological changes associated with TRS are categorically different from those in treatment-responsive schizophrenia or are on a continuum with those seen in treatment-responsive patients.^97^ Thus, a goal of research priorities for TRS should be to differentiate the underlying neurobiological factors among patients with TRS from those in treatment-responsive patients (Fig. 3).

## Conclusions

The roadmap for understanding the pathophysiology of TRS and improving outcomes for patients should focus on developing methods for categorizing this patient group based on features such as the stage of illness when TRS emerges, clinical phenomenology, the response to dopamine antagonists and clozapine, and biomarkers. Two main dopaminergic theories have been proposed to explain TRS. The first proposes that TRS is characterized by normal dopamine function, while glutamate or other pathways contribute to the distinct neurobiology of TRS. The other proposes that dopamine supersensitivity leads to the development of TRS over time. It is important to recognize that these two models are not mutually exclusive. Moreover, they may explain different presentations of TRS, with the normal dopamine hypothesis explaining treatment resistance from illness onset, and the dopamine supersensitivity hypothesis explaining the development of treatment resistance in some patients (Table 2).

**Table 2:** Key points.

Key point	Rationale
First-episode TRS may be neurobiologically distinct from TRS that develops over time^10^	● DSP hypothesis only accounts for TRS in patients who initially responded to an antipsychotic^32,36^
	● Differences in dopamine synthesis capacity in the striatum may be present from illness onset^53,56^
	● Biological changes in TRS may be categorically distinct (i.e., subtypes) from or on a continuum with changes observed in treatment-responsive patients^97^
Development of TRS may occur through multiple pathways	● Genetic susceptibility to TRS is not exclusive to dopamine-related genes^62^
	● Glutamate hypothesis of TRS is supported by
	○ Neuroimaging studies^24,69,70^
	○ The potential involvement of glutamatergic systems in the mechanism of action of clozapine, the only drug approved to treat TRS^15,69^
	● Other hypotheses of TRS suggest that serotonin or inflammation and oxidative stress may be involved^82,84,88,89^
Future work should address limitations to the hypotheses of the neurobiology of TRS	● Inconsistent definitions of TRS
	● Small treatment group sizes
	● Not specific to phase of illness at TRS onset (i.e., first episode vs later development)

*DSP* dopamine sensitivity psychosis, *TRS* treatment-resistant schizophrenia

There are still many gaps in the understanding of the pathophysiology and pathways leading to the development of TRS. Closing these gaps may lead to improved treatment options for patients with TRS. Several candidates for the biological mechanism and/or subtypes of TRS have been identified. However, none have been widely replicated to date, and more research is needed to test them in different patient populations and different phases of illness. The likely heterogeneity of pathways into and mechanisms sustaining TRS within this population further complicates research. Given the major health burden of TRS for patients and families and to make significant progress toward these goals, it would be useful if organizations such as the National Institute of Mental Health (NIMH) in the United States and similar funding bodies around the world placed more emphasis and funding on research into the underlying biology of TRS.

## Methods: search strategy and selection criteria

A selective PubMed search was performed for the dates January 1, 2007, to September 26, 2018, using the following terms: “schizophrenia” AND “drug resistance” OR “treatment refractory” OR “treatment failure” OR “treatment refractoriness,” “clozapine,” “treatment resistant schizophrenia,” “biology of treatment resistant schizophrenia,” “biological mechanisms of treatment resistant schizophrenia,” “pathophysiology of treatment resistant schizophrenia,” “pathways to treatment resistant schizophrenia,” “imaging” AND “treatment resistant schizophrenia,” “PET imaging” AND “treatment resistant schizophrenia,” and “spectroscopy” AND “treatment resistant schizophrenia.” Additional searches were conducted based on reviews of the articles identified in the initial PubMed search. Articles included in this narrative review were limited to those in English-language, peer-reviewed journals.

## Data Availability

No data sets were generated or analyzed for this study.
